# Prevalence and risk factor of post-operative lower extremities deep vein thrombosis in patients undergoing gynecologic surgery: a single-institute cross-sectional study

**DOI:** 10.1186/s12959-022-00376-0

**Published:** 2022-04-04

**Authors:** Supakorn Lorchaivej, Prapaporn Suprasert, Tanop Srisuwan, Jintana Rujiwetpongstorn

**Affiliations:** 1 Division of Gynecologic Oncology, Department of Obstetrics and Gynecology, Faculty of Medicine, Chiang Mai University, Chiang Mai, 50200 Thailand; 2 Department of Radiology, Faculty of Medicine, Chiang Mai University, Chiang Mai, Thailand; 3 Unit of Diagnostic Radiology, Sripat Medical Center, Faculty of Medicine, Chiang Mai University, Chiang Mai, Thailand

**Keywords:** Deep vein thrombosis, Gynecologic surgery

## Abstract

**Background and aim:**

The study of prevalence and risk factors of postoperative lower limb deep vein thrombosis (DVT) in Thai gynecologic patients was limited. The present study was conducted to evaluate this issue.

**Methods:**

The patients were age > 15 years old without a history of DVT or pulmonary emboli (PE) scheduled for laparotomy or vaginal gynecologic surgery between May and November 2020 were invited to participate. All of these patients were scheduled for a complete duplex ultrasound to detect lower limb DVT 72 h before and within 14 days after the operation. The patients without DVT were scheduled for an interview by telephone about DVT symptoms 30 days after the operation. The clinical variables were compared using univariate and multivariate analysis to identify the independent factors related to the development of DVT.

**Results:**

One hundred and twelve patients met the inclusion criteria. Of these patients, 44 cases (39.3%) were diagnosed as malignancy and 102 patients underwent a hysterectomy. Post-operative DVTs were detected in six patients (5.4%) and all except one had a malignancy. Thus, the prevalence of DVT in malignancy cases was five in 44 patients (11.4%). The independent risk factors for postoperative DVT were age > 60-year-old and receiving a perioperative blood transfusion. Five of six DVT patients received low molecular-weight heparin for treatment of DVT and none developed PE. The rest of the participants reported no symptom-related DVTs from the interview 30 days after the operation.

**Conclusion:**

The prevalence of postoperative DVT in gynecologic patients was 5%, and the independent risk factors were elderly patients and receiving a perioperative blood transfusion.

**Supplementary Information:**

The online version contains supplementary material available at 10.1186/s12959-022-00376-0.

## Introduction

Deep vein thrombosis (DVT) is a condition described as one or more blood clots forming in a deep vein mainly in the legs and pelvis. DVT is the most important source of pulmonary embolism (PE) [1]. The etiology of DVT is vascular endothelial damage, the stasis of blood flow, and a hypercoagulation state [2]. Therefore, the risk factors for developing DVT include advanced age, prolonged immobility, malignancy, major surgery, trauma, prior venous thromboembolism, hormonal usage, obesity, chronic heart failure, nephrotic syndrome, inflammatory bowel disease, myeloproliferative disorders, pregnancy, and postpartum period [3]. Postoperative DVT appears more common in Western countries as high as 35% while in Asian countries were varied with a range of 3–28% [4, 5]. Regarding gynecologic surgery in an Asian country, Qu et al. [6] recently reported the incidence of DVT in Chinese patients undergoing gynecologic surgery was 9.20% and Sermsathanasawadi et al. [7] studied the prevalence of perioperative asymptomatic DVT in Thai gynecologic cancer patients with a prevalence of 7%. The high prevalence of DVT in the Western country supported the benefit of prophylactic DVT medication in many guidelines [8] but it is not popular in most Asian physicians including Thailand due to bleeding concerns. Because of a wide range of the prevalence of DVT in postoperative gynecologic surgery in Asia and the limited studies in Thai patients, we conducted this cross-sectional study to evaluate the prevalence and potential risk factors of postoperative lower limb DVT by using complete duplex ultrasound (CDUS) in Thai patients who underwent gynecologic surgery. This knowledge should be a benefit for the decision of using prophylactic DVT medications in this situation.

## Materials and methods

### Patient selection

This cross-sectional study was conducted between May 13, 2020, and November 13, 2020, after approval by the local research committee. The inclusion criteria were as follows; 1) the patients over 15 years old who were scheduled for laparotomy or vaginal gynecologic surgery 2) no history of DVT or PE and 3) available for an ultrasound appointment. The patients with ulcers on their legs or using anticoagulant drugs or who could not communicate well or with a detected preoperative DVT were excluded from the study. None of the patients received any perioperative DVT prophylaxis.

After informed consent, the patients were scheduled for CDUS by each of two board-certified radiologists (JR: 9 years of experience and TS: 15 years of experience). The CDUS of bilateral lower limb veins was performed as described in the standardized approach consensus recommendations [9] with additional scanning of anterior tibial veins and muscular veins of the calf. In short, the procedure included compression ultrasound from the common femoral vein down to tibial veins and color with spectral Doppler of common femoral and popliteal veins. Four ultrasound machines were used in this study: EPIQ5 (Philips Healthcare), iU22 (Philips Healthcare), Aplio500 (Toshiba Healthcare), and Logiq E10 (GE Healthcare) with linear vascular transducers which a frequency bandwidth in the range of 3 to 13 MHZ.

All the participants were scheduled to receive CDUS within 72 h preoperative day and within 14 days after operative days. The patients found to have the DVT during the preoperative period were withdrawn from the study and given further treatment for DVT. In addition, the patients with DVT in the postoperative period received a hematology consultation for optimal treatment of DVT. After 30 days of the operation, all patients who were without DVT after surgery were scheduled for follow-up evaluation of DVT symptoms such as leg pain, leg swelling by one of the investigators (SL) via telephone and. The patients who suspicious DVTs were appointed to confirm the diagnosis by CDUS. The patients unable to receive CDUS within 72 h preoperatively and within 14 days postoperatively for any reason were withdrawn from the study.

The flowing clinical data were collected: age, body mass index (BMI), parity, hormonal usage, chemotherapy, radiation, underlying disease, diagnosis, operation, perioperative blood transfusion, hospital length of stay, and the detail of compression ultrasound findings.

### Sample size calculation

The sample size was calculated using the following formula [10].

$$ \mathrm{n}=\frac{{\mathrm{Z}}_{1-\raisebox{1ex}{$\upalpha $}\!\left/ \!\raisebox{-1ex}{$2$}\right.}^2\mathrm{P}\left(1-\mathrm{P}\right)}{{\mathrm{d}}^2}. $$

where

$$ \alpha =\mathrm{Level}\ \mathrm{of}\ \mathrm{significance}=0.05. $$

$$ \mathrm{Z}=1.96. $$

p meant the population of gynecologic patients who developed postoperative DVT from the previous study was 0.07 [11] while d meant the precision value that equal to 0.05. Then a minimum sample size of 107 patients was needed after included the error of the data at 5%.

### Statistical analysis

Statistical analysis of the data was carried out using IBM SPSS statistics V22.0 for the Window program (IBM, Armonk, New York, USA). Descriptive data were presented as the percentage. Chi-square or Fisher’s exact test was used for comparative analysis of the factors between the non-DVT and DVT patients and to calculate the odds ratio of categorical variables and were also used for comparison between the clinical variables. Binary logistic regression analysis with a backward stepwise method was used to identify the independent potential risk factors for developed postoperative DVT. A *p*-value of < 0.05 was considered statistically significant.

## Results

Two hundred and fifty-six patients were scheduled for laparotomy and vaginal gynecologic surgery in this study. However, 26 cases were refused to participate in the study and 118 patients were excluded and withdrawn due to history of DVT and PE (3 cases), inability to receive compression ultrasound imaging at the scheduled time (111 cases), difficulty with communication (4 cases) and preoperative ultrasound positive for DVT (2 case), Of these 118 cases, 5 patients did not have surgery because they were switched to hormonal treatment [1], observation [1], intervention management [1], and surgery was postponed [2]. As a result, 113 patients were operated on, with laparotomies being performed in 102 cases, the most prevalent procedure being hysterectomy in 69 cases, and vulva operations being performed in the remaining 11 cases. All excluded patients had a mean age of 49.39 + 11.90 years and a mean BMI of 23.31 + 3.71 kg/m2. About 38.1% of them had underlying disease, the most common of which was hypertension. The final pathology confirmed malignancy in 45 patients (39.8%), with ovarian cancer being the most common diagnosis. Only one of the patients who were excluded developed post-operative pulmonary emboli, which were detected by computed tomography angiography (CTA) six weeks after surgery. This patient was 45 years old. She was diagnosed with stage IIIB cervical cancer, bowel obstruction, and uterine perforation. Her operation consisted of bilateral salpingo-oophorectomy, small bowel resection, colostomy, and appendectomy. She developed prolonged postoperative fever without any dyspnea symptoms. The CT-whole abdomen was done at 6 weeks after operation to find out the etiology of fever and found segmental pulmonary artery embolism in the right lower lung. Therefore, computed tomography angiography (CTA) was done after that to confirm the diagnosis. She investigated DVT with CDUS and found nothing. The patient was treated with enoxapalin 0.4 mL subcutaneous injections every 12 h for her PE. The details of these excluded cases were summarized in Appendix 1.

Finally, 112 patients were recruited in this study. The clinical data were presented in Table 1. The mean age was 51.11 years old and the mean BMI was 23.77 kg/m2. Underlying disease was found in 41% of the studied patients. Nearly 40% were diagnosed with malignancy lead by cervical and endometrial cancers. All except two patients received a laparotomy with the most common operation of hysterectomy. About 20% of the patients underwent surgical staging. Twenty-two patients received perioperative blood transfusions in a range of 1–8 units. The mean operative time was 159.13 min.

**Table 1:** Clinical Characteristics (*N* = 112)

	N (%)
Mean age (years)	51.11 + 11.80
Mean BMI (Kg/m2)	23.77 + 3.77
Mean operative time (minutes)	159.13 + 68.29
Mean hospital length (days)	7.7 + 3.2
Single	24 (21.4)
Married	87 (77.7)
Divorce	1 (0.9)
Nulliparity	34 (30.4)
Underlying disease *	46 (41.1)
Hormone usage	16 (14.3)
Current OCP usage	5
Previous OCP usage	8
Previous hormonal therapy usage	3
Chemotherapy	15 (12.8)
Neoadjuvant setting	7
Previous chemotherapy	8
Previous radiation	4 (3.6)
Diagnosis	
Benign	68 (60.71)
Myoma	37
Adenomyosis	11
endometriosis	8
Ovarian tumor	7
Other	5
Malignancy	44 (39.28)
CA ovary	6
CA cervix	12
CA endometrium	12
CA vulva	1
CA fallopian tube	7
Uterine sarcoma	2
Ovarian metastasis	4
Operation	
TAH	1 (0.9)
TAH and adnexal surgery	50 (44.6)
TAH and adnexal surgery and other	16 (14.3)
TAH and bowel surgery	3 (2.7)
Adnexal surgery	5 (4.5)
Surgical staging	23 (20.5)
RHPL	10 (8.9)
Myomectomy	2 (1.8)
Vulva surgery	2 (1.8)
Perioperative blood transfusion	22 (19.6)
1 unit	6 (5.4)
2 unit	11 (9.8)
3 unit	1 (0.9)
4–8 unit	4 (3.6)

*Underlying disease: Diabetes Mellitus [8], hypertension (40), dyslipidemia (32), chronic kidney disease [2], IgA nephropathy [1], cirrhosis [1], SLE [2], systemic sclerosis [3], myelodysplastic syndrome [1], stroke [1], coronary artery disease [1]

BMI = body mass index, OCP = oral contraceptive pill, TAH = total abdominal hysterectomy, RHPL = radical hysterectomy and pelvic lymphadenectomy

Postoperative DVTs were detected in six cases (5.4%) without symptoms of DVT. The median duration of postoperative ultrasonography was 71 h with a range of 42–306 h. The detail of these six patients was summarized in Table 2. All except one were diagnosed with a malignancy. Therefore, the prevalence of DVT in patients with malignancy was 5/44 (11.4%). Four cases were more than 60 years old. Two cases had no underlying disease. The main operation performed was a hysterectomy. The location of DVT was on the left side (two cases), right side (three cases), and both sides in the remainder All DVTs were distal. Five cases received enoxaparin for treatment of DVT. One case refused the DVT treatment, and no symptoms occurred. Regarding adverse effects from anticoagulants, one patient developed vaginal bleeding after 17 days of enoxaparin and discontinued anticoagulant treatment with a dissolved DVT 5 weeks after the operation. None of the six patients developed PE.

**Table 2:** Details of Patients with Postoperative DVT

sn	Age (year)	BMI (kg/m^2^)	UD	Diagnosis	Operation	Operation time (min)	Perioperative blood transfusion (unit)	Duration of US (hours)	Location of DVT	Treatment	Note
26	68	25.5	HT,DLP	CA colon with ovarian metastasis	Interval debulking TAH and BSO and debulking surgery	150	none	72	Left gastrocnemius vessle	Enoxapalin 0.4 ➔ 0.6 ml SC OD	LOS 14 days ➔ lost to follow up
44	67	21.8	HT	Uterine cancer (stage IV carcinosarcoma)	TAH and BSO and debulking surgery	198	2	120	Left soleal vein	Enoxapalin 0.5 ml SC q 12 h 3 mos	LOS 12 days ➔ supportive treatment
51	45	23.2	none	endometrioma	TAH and BSO and lysis of adhesions	224	none	47	Distal right soleal vein	Enoxaparin 0.6 ml sc q 12 h 17 days	LOS 7 days, off enoxaparin due to bleeding per vaginal stump and FU at 5 weeks after that the DVT was disappeared.
56	42	25.0	HT	CA cervix IIIC1(p) Neuroendocrine tumor	RHPL and BSO	195	none	167	Right soleal vein	None Patient refused treatment	LOS 9 days
67	64	24.8	none	CA ovary (Recurrent)	Debulking tumor with bowel resection and repair vaginal stump and repair bladder	411	2	306	Both soleal vein	Enoxaparin 0.6 ml sc q 12 h for 5 weeks ➔ bemiparin 7500 u sc for 6 months	LOS 19 days
111	83	16.2	HT, CAD	CA fallopian tube stage IIIA2	TAH with BSO with ascites collection with omentectomy with rectal Bx	80	none	73	Right soleal vein	enoxaparin 0.4 ml sc od until now	LOS 23 days

*SN* = Serial number, *BMI* = Body mass index, *UD* = Underlying disease, *US* = Ultrasound, *DVT* = Deep vein thrombosis, *HT* = Hypertension, *DLP* = Dyslipidemia, *TAH* = Total abdominal hysterectomy, *BSO* = Bilateral salpingo-oophorectomy, *SC* = Subcutaneous injection, *q* = every, hr. = hours, *LOS* = Length of stay, *RHPL* = Radical hysterectomy and pelvic lymphadenectomy, *CAD* = Coronary artery disease, *Bx* = Biopsy

Table 3 showed the potential risk factors for developed DVT in the postoperative period. We found that age of 60 years or older, malignancy, presence of underlying disease, the operative time longer than 3 hours and receiving perioperative blood transfusion were significant in univariate analysis. However, only age of 60 years or older and receiving perioperative blood transfusion were independent factors in multivariate analysis with the adjusted odds ratio of 6.905 and 8.917, respectively.

**Table 3:** Factors to Predict Deep Vein Thrombosis

Factors	Total (%)	Number of Patients Divided by DVT Status		Univariate Analysis*		Multivariate Analysis†	
		No DVT(%)	DVT (%)	OD (95% CI)	P- Value	Adjusted OD (95% CI)	P- Value
Age (year)				7.636 (1.312–44.434)	0.025	6.905 (1.096–43.522)	0.040
< 60	86 (76.8)	84 (97.7)	2 (2.3)				
> =60	26 (23.2)	22 (84.6)	4 (15.4)				
BMI (Kg/m2)				1.060 (1.012–1.111)	1.0	–	–
> =30	6 (5.4)	6 (100)	–				
< 30	106 (94.6)	100 (94.3)	6 (5.7)				
Benign	68 (60.7)	67 (98.5)	1 (1.5)	8.590 (0.968–76.217)	0.034	3.207 (0.297–34.572)	0.337
Malignancy	44 (39.3)	39 (88.6)	5 (11.4)				
Hormone				1.067 (1.013–1.123)	0.591	–	–
Yes	16 (14.3)	16 (100)	–				
no	96 (85.7)	90 (93.8)	6 (6.3)				
Underlying disease					0.226	1.417 (0.197–10.183)	0.729
No	66 (58.9)	64 (97.0)	2 (3.0)	3.048 (0.534-			
Yes	46 (41.1)	42 (91.3)	4 (8.7)	17.388)			
Hysterectomy				0.942 (0.898–0.988)	1.00	–	–
Yes	103 (92.0)	97 (94.2)	6 (5.8)				
No	9 (8.0)	9 (100)	–				
Operative time				5.310 (0.923–30.567)	0.061	2.101 (0.292–15.138)	0.461
< =3 h	79 (70.5)	77 (97.5)	2 (2.5)				
> 3 h	33 (29.5)	29 (87.9)	4 (12.1)				
Perioperative blood transfusion				9.79 (1.663–57.498)	0.013	8.917 (1.420–55.997)	0.020
No	90 (80.4)	88 (97.8)	2 (2.2)				
yes	22 (19.6)	18 (81.8)	4 (18.2)				

Binary regression: Backward stepwise (conditional) *P* < 0.5

DVT = deep vein thrombosis, BMI = body mass index

All except one (105 patients) of the remaining patients who did not develop DVT were interviewed via telephone about the DVT symptom 30 days after the operation with no symptoms of DVT. One patient who could not be contacted was diagnosed with cervical cancer stage IA2 and underwent radical hysterectomy and pelvic lymphadenectomy with bilateral salpingo-oophorectomy. She was lost to follow up.

## Discussion

CDUS is Thailand’s first-line diagnostic imaging for DVT. It is a useful technique to confirm the presence of DVT because it has a high sensitivity of 94.2% for proximal DVT, 63.5% for isolated distal DVT, and 93.8% specificity [11]. CDUS is often employed when patients develop a clinical suspicion of DVT, such as leg edema. Our institute performs on the CDUS around 225 patients per year. In this study, we did not use laboratory examinations such as d-dimer tests combined with CDUS because we were concerned about false positives of d-dimer tests caused by elderly age, inflammation, pregnancy, malignancy, and surgery [12].

The prevalence of postoperative lower extremities DVT in patients who underwent gynecologic surgery in this study was 5.4%. This prevalence rate was lower than the previous report from China. Qu et al. [6] conducted a retrospective study of the prevalence of DVT detected by CDUS in a cohort of 739 consecutive Chinese patients undergoing gynecologic surgery in both laparotomy and laparoscopic routes. They found the overall prevalence rate of DVT was 9.2%. Of these patients, 2.17% were symptomatic DVT and 7.04% were asymptomatic DVT and most of them were identified with DVT within 7 days after surgery. The non-similar prevalence rate might be from the different study designs, the number of patients, and the type of operation. In the present study, we did not include the patients who underwent laparoscopy. Laparoscopic surgery has been demonstrated to have a greater risk of DVT than laparotomy. This is most likely owing to the high intraoperative pneumoperitoneum pressures employed in laparoscopy, which are known to increase the risk of DVT [13].

Regarding the prevalence of DVT in patients with malignancy, the present study found the prevalence rate increased to be 11.4%. This prevalence rate was higher than the previous report from a Thai study. Sermsathanasawadi et al. [7] reported the prevalence of proximal DVT in 100 gynecologic cancer patients who underwent surgery very low as 2.11%. The DVT was detected by color Doppler duplex ultrasound examination with venous compression test at pre-operative and 7–14 days after the operation. The authors did not mention the route of surgery. This difference might be from the variation of the ultrasound scanning area. In our study, both the proximal and distal legs were scanned to detect the DVT while the former study scanned only the proximal leg area.

The potential independent risk factors of DVT in the present study were the elderly age more than 60 years old and receiving perioperative blood transfusion. The elderly population is usually associated with vascular sclerosis, high blood viscosity, and poor venous valve function. These conditions are believed to increase the rate of DVT [14].

For the perioperative blood transfusion, Hod EA [15] summarized that transfused red blood cells have been postulated to influence the inflammatory process via microparticles from storage RBC, which can have prothrombogenic potential and ultimately potentiate a hypercoagulable condition that might lead to increased risk of DVT. Our study was consistent with a recent study from North America, in which perioperative blood transfusion was linked to an elevated risk of postoperative DVT and PE, with adjusted odds ratios of 2.2 and 1.9, respectively [16].

Regarding malignancy, patients who received gynecologic cancer surgery were considered to be at high risk of DVT due to advanced age, cancer type, presence of pro-thrombotic mutations, pelvic mass compressing the major pelvic veins, endothelial cell injury during pelvic lymphadenectomy, lengthy surgical procedures, and thrombogenic chemotherapy [17, 18]. In the present study, five from six patients were diagnosed with a malignancy. However, malignancy status was not the independent factor for developing DVT in our study. This might be from an inadequate number for detecting significance in multivariate analysis.

Concerning the location of DVT, our study found five of six cases developed DVT at the sole vein located in the calf area. This site is the most common location from a previous study [19]. Yoshimura et al. [19] conducted a retrospective study to evaluate the most common site of DVT in 137 Japanese patients who were diagnosed with DVT or PE by Computed tomography pulmonary angiography (CTPA) combined with venous-phase imaging of lower extremity [CT venography (CTV)]. The authors found the muscular veins composed of the social and gastrocnemius were the most common site of DVT right, left, or both sides. Another large cohort study from Europe, Maeseneer et al. [20] reported the anatomical site in 1338 patients who were diagnosed with acute lower limb DVT detected by duplex ultrasound method. They found the left-sided DVT to be predominant. In 443 patients who developed DVT limited to one segment, 370 patients (83.5%) had DVT isolated to the calf veins. One explanation for the predominance of DVT on the left side is possibly from the frequent compression of the left common iliac vein by the overriding right common iliac artery [21]. However, from six patients who developed DVT in the present study, The location on left, right, and both sides were two, three, and one case, respectively. The non-predominate left-side DVT in our study might be from the low occurrence number.

The relationship of the DVT at soleal vein and PE was investigated in the previous study. Ro and Kageyama [22] studied 100 autopsy cases with PE and lower limb DVT by examined all deep venous segments that included iliofemoral vein, popliteal vein, and soleal vein. The result showed that the soleal vein had the highest incidence of DVT in all venous segments. The authors concluded that the soleal vein was considered to be the primary site of DVT and further propagated to the proximal veins through the drainage veins. In the present study, the location of DVT in all six patients was calf vein that hematologists advised to treat with low molecular weight heparin (LMWH). However, one patient refused this treatment and the other two patients discontinued LMWH 2 weeks due to vaginal bleeding in one case and lost to follow up in the other one. Fortunately, all of them did not develop PE. The duration of anticoagulant treatment for DVT according to the standard guidelines was at least 3 months [23].

The strength of the present study was the cross-sectional design study in that the clinical data were recorded in a prospective setting. This decreased the recall bias data. In addition, the compression ultrasound was performed by experienced ultrasonologists, therefore the accuracy of the DVT results was very high. However, at 30 days postoperative time, we did not investigate all the participants again, though we could not detect the asymptomatic DVT. We knew only symptomatic DVT did not occur in contactable precipitants.

## Conclusion

The prevalence of lower limb DVT in postoperative gynecologic patients was 5.4% and increased to 11.4% in gynecologic cancer patients. The independent potential risk factor for DVT was the advanced age more than or equal to 60 years old and the receiving of perioperative blood transfusion. In elderly gynecologic cancer patients and those who received a perioperative blood transfusion, DVT prevention strategies should be considered.

## Supplementary Information

**Additional file 1.**

## Data Availability

The datasets used and/or analyzed during the current study are available from the corresponding author on reasonable request.

## Abbreviations

DVT: Deep vein thrombosis
PE: Pulmonary emboli
CDUS: Complete duplex ultrasound
LMWH: Low molecular weight heparin
