# Supplementary material for: Prevalence and risk factor of post-operative lower extremities deep vein thrombosis in patients undergoing gynecologic surgery: a single-institute cross-sectional study
Source: Thromb J. 2022 Apr 4;20:14. doi: 10.1186/s12959-022-00376-0 (PMC8978349; doi:10.1186/s12959-022-00376-0)
Supplement: Supplementary file 1 — Additional file 1. [file 12959_2022_376_MOESM1_ESM.docx]

Appendix 1

Excluded 144 cases

Denied to participate the study = 26 cases

Data available = 118 cases

No surgery in 5 cases due to change to hormonal treatment (1), change to observe (1), intervention management (1), and postponed surgery (2).

Surgery 113 cases

Table X: The clinical data of excluded patients who underwent operations

|  | N (%) |
| --- | --- |
| Mean BMI (kg/m2) | 23.31+3.71 |
| Mean age (year) | 49.39+11.90 |
| Postoperative DVT | 1 * |
| Preoperative DVT | 5 |
| Underlying disease** | 43 (38.1) |
| Surgery  Vulva surgery  Explore lap  TAH +/- bilateral salpingectomy  TAH +/- adnexal surgery  Complete surgical staging  Radical hysterectomy + BPND  Myomectomy  BPND | 11(9..7)  102(90.3)  13(11.5)  35(31.0)  13(11.5)  8(7.1)  20(17.7)  1(0.9) |
| Benign  Myoma  Adenomyosis  Endometriosis  Myoma + adenomyosis  HSIL  Ovarian tumor  Uterine tumor  Vaginal mass  Pelvic organ prolapse | 68(60.2)  41(36.3)  4(3.5)  6(5.3)  4(3.5)  1(0.9)  7(6.2)  1(0.9)  1(0.9)  3(2.7) |
| Malignancy  CA ovary  CA cervix  CA endometrium  CA vulva  CA tube  CA colon  Borderline tumor of ovary  Primary peritoneal adenocarcinoma | 45(39.8)  14(12.4)  12(10.6)  8(7.1)  4(3.5)  1(0.9)  1(0.9)  4(3.9)  1(0.9) |

*This case was excluded due to inability to receive compression ultrasound imaging at the scheduled time

**Underlying disease:Hypertension (HT) (11), Malignant melanoma (1), Systemic lupus erythematosus(SLE) (1), diabetes mellitus (DM) +DLP (1),HT+DLP(6),CA breast (5),valvular heart disease (1), DM+HT+DLP+CA breast (3), DM+HT (2), Thyrotoxicosis (5), epilepsy(1), DLP(3),HT+coronary artery disease (CAD) (1), DM (2)

BMI=body mass index, DVT=deep vein thrombosis,TAH=total abdominal hysterectomy,BPND=bilateral pelvic node dissection,HSIL=high grade squamous cell intraepithelial lesion,
